# *Pseudomonas fluorescens* HK44: Lessons Learned from a Model Whole-Cell Bioreporter with a Broad Application History

**DOI:** 10.3390/s120201544

**Published:** 2012-02-06

**Authors:** Josef Trögl, Archana Chauhan, Steven Ripp, Alice C. Layton, Gabriela Kuncová, Gary S. Sayler

**Affiliations:** 1 Faculty of the Environment, Jan Evangelista Purkyně University in Ústí nad Labem, Králova Výšina 3132/7, 400 96 Ústí nad Labem, Czech Republic; 2 The Center for Environmental Biotechnology, The University of Tennessee, 676 Dabney Hall, Knoxville, TN 37996, USA; E-Mails: achauha1@utk.edu (A.C.); saripp@utk.edu (S.R.); alayton@utk.edu (A.C.L.); sayler@utk.edu (G.S.S.); 3 Institute of Chemical Process Fundamentals of ASCR, v.v.i., Rozvojová 135, 165 02, Prague 6-Suchdol, Czech Republic; E-Mail: kuncova@icpf.cas.cz

**Keywords:** bioluminescence, bioreporter, biosensors, *Pseudomonas fluorescens* HK44, *lux* genes

## Abstract

Initially described in 1990, *Pseudomonas fluorescens* HK44 served as the first whole-cell bioreporter genetically endowed with a bioluminescent (*luxCDABE*) phenotype directly linked to a catabolic (naphthalene degradative) pathway. HK44 was the first genetically engineered microorganism to be released in the field to monitor bioremediation potential. Subsequent to that release, strain HK44 had been introduced into other solids (soils, sands), liquid (water, wastewater), and volatile environments. In these matrices, it has functioned as one of the best characterized chemically-responsive environmental bioreporters and as a model organism for understanding bacterial colonization and transport, cell immobilization strategies, and the kinetics of cellular bioluminescent emission. This review summarizes the characteristics of *P. fluorescens* HK44 and the extensive range of its applications with special focus on the monitoring of bioremediation processes and biosensing of environmental pollution.

## 1. Introduction

Bioreporters are genetically engineered cells (predominantly microbial) that harbor genetic fusions between responsive gene promoters and reporter genes. Bioreporters enable monitoring of metabolic gene expression or repression via measurement of the signal resulting from the joint expression of the promoter and reporter genes. Reporter genes can code for any easy-to-measure signal. The majority of bioreporters utilize genes for enzyme activities linked to the generation of light, for example, fluorescence (green fluorescent protein (GFP) and its differently colored variants) or bioluminescence (bacterial (*lux*) or insect (*luc*) luciferases) signaling outputs (see reviews [1–3] for details; also see Table 1). According to the type of reporter gene regulation used, bioreporters can be classified into two groups. Metabolic (constitutive) bioreporters express reporter genes constitutively regardless of external inducing or repressing factors. A measured decrease in signal strength indicates that the bioreporter has in some way been injured, typically due to a toxic interaction with its environment, resulting in a change in metabolic status that results in an analogous change in bioluminescent emission. Catabolic (inductive) bioreporters express their reporter genes upon external activation by chemical, physical or biological inducers, and therefore increase their light emission intensity in relation to the concentration of inducer present. Catabolic bioreporters have a wide range of applications in sensing and monitoring strategies, and can be designed to either nonspecifically or specifically report on inducer interactions. In nonspecific applications, the bioreporter responds via an increase in signal linked to general stress or DNA damage effects related to exposure to a cytotoxic, genotoxic, or mutagenic chemical or physical interaction. Although the specific identity of the chemical/physical agent cannot be established, these bioreporters quickly, efficiently, and inexpensively warn of potential threats or harmful incursions that can then be characterized more fully by other analytical methods. If the bioreporter is specific, its increase in emission intensity can be linked directly to a targeted inducer or closely related class of inducers. The *Pseudomonas fluorescens* HK44 bioreporter, for example, emits increased bioluminescence when exposed to naphthalene, salicylate, and related structural analogs [4,5]. Strain HK44 serves as the earliest example of *lux*-based bioluminescent bioreporter technology being implemented within a bacterial host directly isolated from a chemically contaminated ecosystem. Its introduction in 1990 has spurred a long history of novel bioreporter applications and fundamental and applied studies conveying the potential value of bioreporters in environmental monitoring scenarios. The extensive and divergent use of this model living bacterial bioreporter will be examined in this review to provide a critical overview of the advantages and disadvantages of bioreporter sensing and monitoring technologies.

## 2. Characterization of *P. fluorescens* HK44

**Figure 1.**
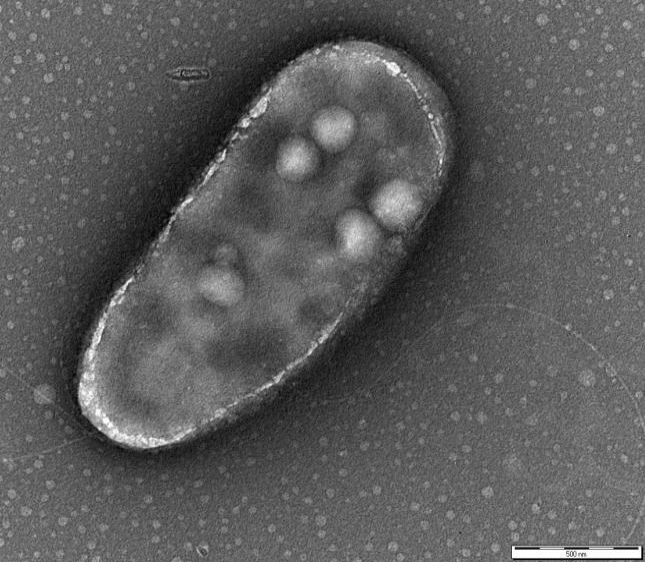
Transmission electron micrograph of *Pseudomonas fluorescens* HK44 encapsulated in a silica gel (reprinted from [38] with permission).

*P. fluorescens* belongs to the Gamma-proteobacteria class and is a common genus of soil and water. The cells are rod-shaped, Gram-negative, ∼2 × 0.5 μm sized, with two bunches of long flagella on both poles (Figure 1). The metabolism is strictly respiratory and predominantly aerobic.

**Figure 2.**
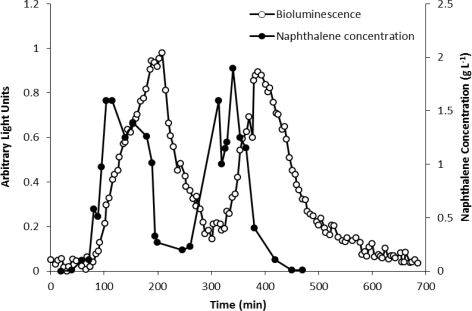
Bioluminescence emission from *P. fluorescens* HK44 in a flowcell exposed to cyclic perturbations of naphthalene. Adapted from [39].

*P. fluorescens* strain HK44 is a genetically engineered strain that responds to exposure to naphthalene, salicylate and other structural analogs by production of visible light [4]. This feature is encoded on the engineered plasmid pUTK21 harboring a *nah-luxCDABE* genetic fusion. The construction takes advantage of the positive induction of the *nah* and *sal* operons by intermediates of the naphthalene metabolic pathway salicylate [4]. Induction of the gene fusion results in expression of the bioluminescent *luxCDABE* genes and subsequent bioluminescent response at an emission wavelength of 490 nm (Figure 2).

### 2.1. Construction of the HK44 Bioreporter

**Figure 3.**
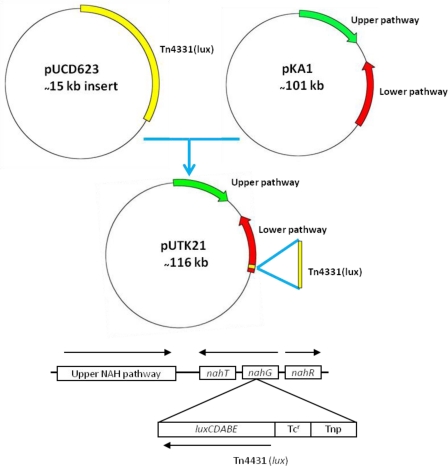
Plasmid pUTK21 contains a transposon-based *luxCDABE* insert positioned within the *nahG* gene. This permits direct observation of naphthalene catabolic activity via emission and real-time measurement of bioluminescence. Tc^r^, tetracycline resistance gene; Tnp, transposase.

Strain HK44 was constructed in two steps (Figure 3). In step 1, the bacterium, *P. fluorescens* 5R (*nah^+^* *sal^+^*), containing the natural plasmid pKA1 harboring the upper (*nahABCDEF*) and lower (*nahGHIJK*) pathways of naphthalene metabolism, was isolated from a manufactured gas plant (MGP) soil [4]. Plasmid pKA1 was transposon mutagenized using the *luxCDABE*-containing transposon Tn4431 carried on the suicide vector plasmid pUCD623. The *luxCDABE* genes on Tn4431 were derived from *Vibrio fischeri* strain MJ-1, which was isolated from the light organ of the fish *Monocentris japonicus* [40]. Tn4431 also contains genes from Tn5 and *E. coli* strain D1021 [41]. The resulting recombinant plasmid was designated as pUTK21 and the bioluminescent construct containing pUTK21 was designated as *P. fluorescens* strain 5RL (*nah^+^* *sal*^−^ *lux^+^*). Strain 5RL was able to produce strong inducible light upon induction by naphthalene or salicylate, but lost its ability to degrade naphthalene completely due to interruption of the *sal* operon by the *lux* gene insertion event [4]. In step 2, plasmid pUTK21 was transferred by conjugation to another wild-type strain, *P. fluorescens* 18H (*nah*^−^ *sal^+^* *lux*^−^), also isolated from a contaminated MGP site. The resulting tetracycline resistant transconjugant had the desired *nah^+^* *sal^+^* *lux^+^* phenotype and was designated as *P. fluorescens* strain HK44. Table 2 provides a summary of the bacterial strains used to construct strain HK44.

### 2.2. Genetic Characterization of Strain HK44

Recently, whole genome sequencing of *P. fluorescens* strain HK44 was performed using the Roche 454 Life Sciences GS FLX system [42]. The shotgun sequences were assembled into 131 contigs that were further compiled into 21 scaffolds using paired-end information. Out of the 21 scaffolds, four contained sequences belonging to plasmids. One of these scaffolds was identified as the recombinant megaplasmid pUTK21 and the remaining three as part of one or more cryptic plasmids. The pUTK21 scaffold had four gaps which were closed by PCR amplification followed by Sanger sequencing resulting in a ∼116 kb plasmid described in more detail below. The unclosed draft genome has a size of ∼6.1 Mb and contains 5,720 protein coding sequences. It has a gene coding density of 84.82%, a G+C mol% of 58.73%, and contains 3 rRNA operons, 56 tRNA genes, and one integrated phage. Putative functions could be assigned to 4,558 of the 5,720 protein coding genes, with 1,617 of them connected to KEGG (Kyoto Encyclopedia of Genes and Genomes) pathways. The HK44 genome shows a range of metabolic potentials including enzymes for multiple carbon source utilization pathways, for the degradation of organic substances including polycyclic aromatic hydrocarbons (PAHs) and other xenobiotic chemicals and numerous ABC-type transporters for organic compound uptake. The genome also contains gene clusters possessing genes for ammonification and nitrogen respiration (*nar*, *nir*, *nas*, and *nor*), which indicates that strain HK44 can carry out nitrate/nitrite reduction by both assimilatory and dissimilatory means. However, owing to the absence of nitrous oxide reductase and nitrogenase genes, strain HK44 is not capable of carrying out denitrification of nitrates and nitrites to dinitrogen.

Thirty percent of the HK44 genomic traits are unique and distributed among five genomic islands, a prophage, and plasmids. They encode pathways conferring novel traits to strain HK44 including a polysaccharide production pathway and a potentially undescribed aromatic carbon degradation pathway. Surprisingly, several virulence factors not characteristic of other *P. fluorescens* strains were identified including a type III secretion system and *P. syringae*-like pathogenicity genes. The combinations of these features supports strain HK44’s ability to thrive both in soil and possibly in commensal relationship with plants [43].

### 2.3. Genetic Characterization of Plasmid pUTK21

**Figure 4.**
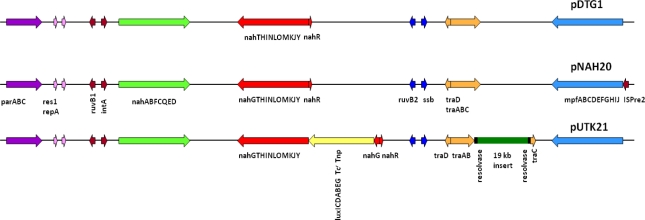
Physical organization of the gene clusters in three plasmids pDTG1, pNAH20, and pUTK21 belonging to *Pseudomonas* strains. Genes of the same color indicate corresponding orthologous genes with high homology (>80%) at the nucleic acid level and arrows indicate direction of transcription (Adapted from [45]).

Preliminary sequencing results showed that the recombinant pUTK21 plasmid of strain HK44 has a total genome size of ∼116 kb with a coding density of 84.01%, G+C mol% of 54.97%, and 135 protein coding genes, among which 82 could be assigned successfully to clusters of orthologous groups of proteins (COGs). Analysis of the upper and lower naphthalene degradation operons, which occupy 9.5 kb and 13 kb regions, respectively, showed that the catabolic genes were identical to those of the IncP-9 naphthalene plasmid pDTG1 from *P. putida* strain NCIB 9816-4 [44] and pNAH20 from *P. fluorescens* strain PC20 [45] (Figure 4). The upper catabolic pathway, which converts naphthalene to salicylate, is located from base-pairs 14,536 to 24,078, while the lower naphthalene catabolic pathway for the conversion of salicylate to acetyl coenzyme A and pyruvate is situated from base-pairs 41,482 to 67,006. The lower salicylate pathway encoded on pUTK21 is interrupted by insertion of the *luxCDABE* gene cassette within the *nahG* gene at position 49,456. However, strain HK44 carries a copy of the salicylate operon on its chromosome (inherited from the 18H wild-type strain) and is therefore capable of mineralization of salicylate and complete mineralization of naphthalene [4]. Both operons are positively inducible by the *nahR* gene product [46], a member of the *lysR* family [47]. In addition, the gene for the methyl-accepting chemotaxis protein for naphthalene, *nahY*, has been identified at the end of the salicylate degradation operon at base-pairs 39,629 to 41,245. The transfer functions of pUTK21 are encoded in a 20 kb segment of the plasmid backbone, typical of the pNAH20 and pDTG1 plasmids, that includes the *tra* transfer genes and the *mpf* mate pair formation genes. However, pUTK21 differs from the pDTG1 plasmids in that the *tra* operon is interrupted by an additional 19 kb DNA insertion that contains homologues of genes required for large plasmid replication, maintenance, and conjugation and chemotaxis (Figure 4). This fragment is flanked by an invertase and site specific recombinases suggesting an evolution that involved the lateral transfer of DNA between bacterial species.

## 3. Biosensing with *P. fluorescens* HK44

Although the original objective for the development of strain HK44 was for environmental biomonitoring of PAH metabolism, it became clear that a biosensor or a bioassay utilizing HK44 could be used for the detection and biosensing of naphthalene, salicylate, and related compounds regardless of their metabolic potential, and within sample matrices besides soil. Thus, researchers over the past 20 years have integrated strain HK44 into a wide variety of fundamental and applied investigational enterprises (Table 3).

### 3.1. Characterization of the HK44 Bioluminescent Response

Due to positive regulation of the *nah* genes, enzymes of the naphthalene degradation pathway as well as *lux* pathway enzymes are always present in low quantities in the HK44 cell resulting in the emission of low background basal levels of bioluminescence even in the non-induced state [48]. Upon induction, after a lag-period, bioluminescence increases due to increased joint expression of the *nah* and *lux* genes. Induction by salicylate occurs directly upon binding to the regulatory NahR protein [46,49]. Consequent interaction of NahR with the *nah* promoter and RNA-polymerase results in increased initiation of transcription of the *nah/sal* operons. Induction by naphthalene occurs indirectly after it is metabolized to salicylate by enzymes of the upper naphthalene degradation pathway. After reaching a maximum, bioluminescence tracks along a slightly lower plateau. Elimination of the inducer leads to a decrease in bioluminescence due to degradation of the luminescence enzymes. The kinetic values for these processes have been determined for both immobilized [39] and suspended [50] cells. The general pattern of bioluminescence kinetics, especially in terms of response time, is variable under different study designs. Flow-through set-ups, where the bioreporter cells remain in a liquid culture, will respond faster than bioreporter cells in an encapsulated state due to the encapsulation matrix somewhat impeding diffusion of the target analyte to the bioreporter. The physiological state of the bioreporter is also critical, with bioreporters maintained under optimal environmental conditions able to respond faster than those that are not. Bioluminescence from strain HK44 is, however, generally repeatable in the range of ±20% [4,51,52].

**Figure 5.**
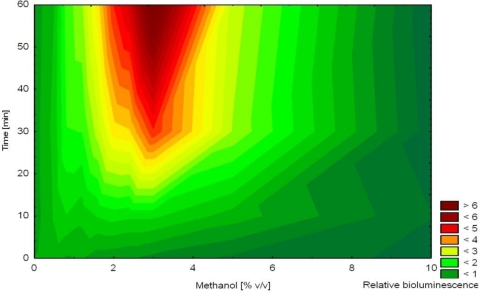
Effect of methanol on bioluminescence emission from non-induced *P. fluorescens* HK44. Values are expressed relative to a control not exposed to methanol. Reprinted from [38] with permission.

The bioluminescence reaction comprises a cyclic oxidation of a long-chain aldehyde (luciferin) by oxygen under catalysis by the luciferase enzyme (*luxAB*) and reduction of formed fatty acid catalyzed by the reductase complex (*luxCDE*) [24–27]. To produce bioluminescence, the cell must be intact and catabolically active to provide sufficient energy and reducing equivalents for bioluminescence [3]. From a physiological point of view, the complex bioluminescent reaction might be affected by various factors that should be considered when evaluating bioluminescence output from bioreporters and biosensors. The bioluminescent process is obligatory aerobic and requires sufficient oxygen and a carbon substrate supply [26,53]. Optimal bioluminescence from strain HK44 is achieved at pH 6–7 [53]. An exponentially growing HK44 culture emits higher non-induced (background) bioluminescence and hence the relative increase upon induction is lower as compared to stationary or starving cultures [48,54]. Bioluminescence also decreases upon interaction with toxic chemicals, including inducers of *nah-lux* genes in strain HK44 [54–57]. It was also demonstrated that HK44 bioluminescence increases upon exposure to organic solvents [54,58]. This effect is known from other bioluminescent assays [59] and is ascribed to membrane perturbations which, through production of increased levels of free fatty acids, leads to an increased supply of aldehyde substrates for luciferase that are normally limited, although this remains hypothetical. At high concentrations of perturbing chemicals, the negative effect prevails leading to a decrease in bioluminescence (Figure 5) [54–57].

### 3.2. Profiling of the Bioluminescent Response to Naphthalene and Salicylate

The analytical potential of strain HK44 was originally demonstrated in the introductory work of King *et al.* [4] where HK44 cells under continuous culture chemostat growth (minimal media + succinate + yeast extract + tetracycline) responded to naphthalene addition (200 μg·L^−1^ at steady state) within 15 min and reached a maximum within ∼4 h. They also showed that the bioluminescent response was cyclic under repetitive on/off naphthalene feed rates, with the 15 min lag time in bioluminescence induction being highly reproducible. In *in situ* soil slurry experiments (1:1 soil:HK44 suspension) using native PAH contaminated soils containing 1,240 mg naphthalene kg^−1^ and soils artificially contaminated with naphthalene at 1,000 mg·kg^−1^, lag times in bioluminescence induction increased to 1 h, which may have been due to quenching of the bioluminescent signal within the turbid slurry matrix.

Burlage *et al.* [60] compared the response of three bioluminescent strains, *P. fluorescens* HK44, *P. putida* RB1351 harboring the *nah-lux* fusion in the upper naphthalene pathway, and *P. putida* RB1401 harboring the *xyl-lux* construct for detection of toluene degradation, to soils from a site contaminated with aromatic hydrocarbons. The samples were mixed 1:1 directly with the cell suspension in mineral media. Bioluminescence of strain HK44 peaked after ∼30 min with an insignificant lag and enabled distinguishing of contaminated and uncontaminated samples. The response of *nah-lux* strain RB1351 was comparable to HK44 and both assays were concluded to be equal.

The biosensing properties of strain HK44 in response to both naphthalene and salicylate bioavailability were studied in more detail by Heitzer *et al.* [48]. In batch assays with free cells in minimal media, the exponentially growing cultures exhibited higher specific bioluminescence than resting cultures. The resting cultures also responded more significantly to non-inducing compounds serving as carbon sources than the exponentially growing cells. Bioluminescence measured 1 h after induction followed a saturation-type dependence on naphthalene and salicylate concentration. The linear range enabling calibration was 0.72 μg·L^−1^ to 3.25 mg·L^−1^ for naphthalene and 0.4 mg·L^−1^ to 20 mg·L^−1^ for salicylate. The lower detection limit for naphthalene was calculated at 0.045 mg·L^−1^ (45 ppb). Comparison of the assay responses to the same amount of salicylate in minimal media, a minimal media soil extract, and soil slurries revealed that in soil extracts the assay could be considered quantitative while in slurries only qualitative. In this case, the detected bioluminescence was ∼1 order of magnitude lower due to light quenching. Only ∼4% of naphthalene was extracted from amended soil using minimal media likely due to sorption onto soil. Nevertheless, the resulting naphthalene concentration in the extract was well estimated by the assay.

The observation that bioreporter measurements of naphthalene concentrations are lower than concentrations derived by solvent extraction and chemical analysis are due to the fact that naphthalene is highly sorbed onto soil particles and thus it is not bioavailable to the bacteria for biotransformation [73]. In the mid-2000s a series of studies was performed that indicated biosensor performance can be improved using a non-exhaustive extraction technique (NEET) [73]. In addition, this approach allowed for a determination of biodegradation potential of a PAH contaminated soil sample [58,73,79,80]. The main criteria for NEET to be compatible with biosensors is that the solvent should be primarily aqueous or if the extract is in an organic solvent it must be dilutable in an aqueous solution [79]. Initially, several chemicals were tested for their ability to extract PAHs from contaminated soil followed by testing in bioassays, including cyclodextrin (hydroxylpropyl-β-cyclodextrin), Amberlite XAD-4, and organic solvents (methanol and dimethylsulphoxide) [73]. Of these compounds, cyclodextrins, which consist of a hydrophobic cavity and a high aqueous solubility, were proven to be the most effective in extracting naphthalene from soils followed by either mineralization studies or coupling with biosensors [79]. Thus, coupling of the HK44 bioreporter with NEET improves the ecological relevance for determining the bioavailability of PAHs in historically contaminated soils.

Heitzer *et al.* [54] focused on the external factors affecting bioluminescent determination of naphthalene in complex real samples using the HK44 assay. While the presence of toxic admixtures resulted in a decrease in bioluminescence and underestimation of naphthalene concentrations, response to JP-4 complex fuel mixtures coincided with naphthalene concentration but overestimated it. The latter effect was ascribed to membrane perturbations. Significant overestimation of PAH concentration by the HK44 assay (1:1 cell suspension + sediment) compared to gas chromatography analysis (GC-MSD) was also observed by O’Neill *et al.* [69] in contaminated marine sediments. The HK44 assay was capable of discrimination between contaminated and uncontaminated sediments; however, the quantitative response was inaccurate.

### 3.3. Development of Coupled HK44-Bioluminescent Measurement Biosensors

The first HK44 biosensor was based on immobilized HK44 cells in approximate 6 mm diameter alginate beads [39], and the induction kinetics in a packed bed reactor exposed to naphthalene or salicylate at various flow rates was studied. The alginate beads exhibited favorable transport and adsorption properties but as demonstrated by Ripp *et al.* [62] in actual field experiments, biosensors containing alginate encapsulated cells were prone to desiccation after only a few days. This group next designed a flow-through biosensor that utilized HK44 cells encapsulated in strontium alginate on an optical light guide, which allowed direct immersion of the bioreporters into the sample matrix for on-line, real-time measurement of resulting bioluminescence [51]. The practical utility of this biosensor set-up was tested in a waste stream effluent originating from a water solution saturated with JP-4 jet fuel (final effluent naphthalene concentration of 0.55 mg·L^−1^) or an aqueous leachate from an MGP soil (final leachate naphthalene concentration of 0.6 mg·L^−1^). The HK44 biosensor responded to JP-4 jet fuel effluent and the MGP leachate within approximately 22 min, which demonstrates excellent response characteristics under relatively authentic real-world conditions. However, bioluminescent output under these exposure conditions was significantly less than that observed under similar concentration exposures to pure naphthalene, indicating that other components within the complex mixtures were likely contributing toxic or quenching effects.

Valdman *et al.* [71] constructed a flow-through sensor for detection of naphthalene in water consisting of a transparent 0.4 mL flowcell interfaced to a photodetector. The response time after naphthalene introduction at 0.5 mg·L^−1^ was 19 min with maximum bioluminescence being produced within 35–40 min. Naphthalene concentration calibrated against specific bioluminescence was linear up to 0.4 mg·L^−1^. The observed detection limit of 0.02 mg·L^−1^ was below the Environmental Protection Agency (EPA) limit for naphthalene in drinking water (0.1 mg·L^−1^). The HK44 concentration yielding the optimal bioluminescent signal was estimated to be 0.2 g·L^−1^ wet weight, which agreed with the model developed by Uesugi *et al.* [64] showing the bioluminescent response from HK44 to be highly dependent on cell concentration. In a subsequent study by this group, HK44 was used as a bioreporter in the influent and effluent of a petroleum industry’s wastewater treatment system containing 2.4 and 1.1 mg L^−1^ of total hydrocarbons, respectively [72]. Bioluminescence corresponded to hydrocarbon concentrations including the precise estimation of naphthalene, however, at lower dilutions a decrease in the specific bioluminescence occurred likely due to sample toxicity. Because the water also contained nitrate, its effect on the bioluminescent response at 0.5 mg·L^−1^ naphthalene was evaluated. A nitrate concentration of 15 mg·L^−1^ caused an approximate doubling of specific bioluminescence compared to controls without nitrates, while nitrate concentrations of 30 mg·L^−1^ and higher caused a significant decrease in bioluminescence. It was hypothesized that at lower nitrate concentrations, nitrate respiration renders more available oxygen for the bioluminescent reaction, thus increasing bioluminescence emission intensity. However, at higher nitrate concentrations the toxicity of nitrates prevailed leading to decreased bioluminescence.

Trögl *et al.* [52] encapsulated stationary-phase HK44 into thick silica films prepared from prepolymerized tetramethoxysilane (TMOS). The cell concentration was optimized to 10^7^ cells·g^−1^. At ambient temperature, the bioluminescent response from the films upon salicylate/naphthalene induction had a ∼50 min lag and reached a maximum after ∼4.5 h. Bioluminescence exhibited a saturation-type dependence on inducer concentration enabling definition of a linear calibration curve in the range up to ∼5 mg·L^−1^ and lower detectable concentrations of 0.05 and 1.2 mg·L^−1^ for salicylate and naphthalene, respectively. The bioluminescent response was stable for at least 8 months under refrigerated storage.

Valdman and Gutz [78] constructed a flow-through biosensor for the detection of naphthalene in air. The system utilized late-exponential phase HK44 cells (10^7^ cells·g^−1^ encapsulated in 2% agar with 0.01% yeast extract). Upon induction, bioluminescence followed a 30 min lag time and reached a maximum after 80 min. The linear response of the biosensor was in the range from 0.006–0.03 mg·L^−1^ (50–260 nmol·L^−1^) with a detection limit of 0.003 mg·L^−1^ (20 nmol·L^−1^), which is well below the EPA threshold limit value of 0.94 mg·L^−1^ (7.4 μmol·L^−1^) for naphthalene in air. It was demonstrated that the bioluminescent response was dependent solely on naphthalene concentration and not on the air flow-rate.

### 3.4. Selectivity of the Bioluminescent Response for Naphthalene and Salicylate

An ideal analytical procedure is selective, responding only to the target compound and ignoring other admixtures. However, this is not standard behavior for bioreporters such as HK44 (and likely many others, for example, see Kuncova *et al.* [81]). In theory, the *nahG-luxCDABE* operon might be induced by any compound capable of effectively binding to the NahR regulatory protein or by compounds that are metabolized to such an inducer [55]. This presumption is raised by the fact that *nah* operons are naturally induced by the salicylate intermediate of metabolism and by the convergent nature of catabolism. The pathways of chemical metabolism encoded by this genetic pathway were determined by LeBlond *et al.* [82] who demonstrated the ability of the proteins encoded by the *nah* operon carried on the pUTK21 plasmid in strain 5RL to transform methyl- and methoxy-substituted naphthalenes to corresponding hyroxylated products [82]. Many compounds capable of inducing bioluminescence in strain HK44 were subsequently revealed, including salicylate analogs (e.g., 2-aminobenzoic acid [52]), substituted salicylates (e.g., methylsalicylate [43]), substituted naphthalenes (e.g., naphthalene-1-amine [55]), and higher PAHs [65]. Nevertheless, the majority of these compounds induced lower bioluminescence as compared to the “natural” inducers naphthalene and salicylate [52,55,56,65]. The obtained data were also generalized into structure-activity relationships that were both quantitative [65] and qualitative [56]. Unsurprisingly, toxicity of tested aromatics was best estimated/predicted by log P_O/W_ [56,65]. Substituted naphthalenes with –OH groups were more likely to be toxic to HK44 cells [56]. Induction of bioluminescence was best predicted by lowest unoccupied molecular orbital (LUMO) energy [65]. Mono-substituted and 1,2-disubstituted naphthalenes were more likely to be inducers of bioluminescence in HK44 cells [56].

In a comparative study of six assays utilizing various bioluminescent bioreporters, the response of the HK44 “aromatic” assay to various oils containing predominantly aliphatic hydrocarbons was negligible in contrast to assays utilizing bioreporters inducible by aliphatic hydrocarbons. This comparison confirmed the aromatic group-selectivity of the HK44 response [58].

### 3.5. Monitoring of Bioremediation

**Figure 6.**
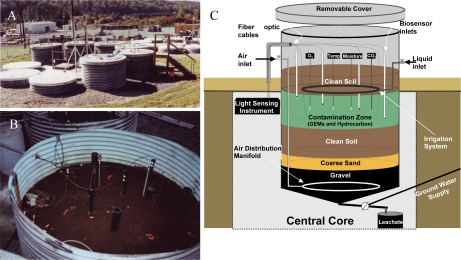
Scheme of the lysimeter facility used in long-term field biodegradation experiment (**A**) The lysimeter facility consisted of six replicate and control soil ecosystems; (**B**) Inside view of one of the six soil packed lysimeters; (**C**) Representative schematic of one of the 4 m deep × 2.5 m diameter lysimeters used for the release of *P. fluorescens* HK44.

With laboratory-based studies demonstrating that strain HK44 could successfully measure naphthalene bioavailability, a natural ecosystem experiment was designed to truly test its biosensing capabilities. Thus, in 1996 strain HK44 became the first genetically engineered microorganism to be approved for field release for applications related to subsurface soil bioremediation [14,61,62,83]. Bioremediation is considered one of the most economically effective treatment technologies available for site clean-up, but ancillary costs associated with monitoring the progress of bioremediation, typically using chemical analysis methods such as gas chromatography/mass spectrometry (GC/MS), increase costs considerably. Using HK44, it was theorized that its bioluminescent signaling in response to PAH contaminants could be applied as a continuous on-line monitoring tool to gauge the effectiveness and endpoints of the bioremediation process. This, of course, requires that the HK44 microbes remain viable throughout the bioremediation effort. Genetically engineered microorganisms are widely assumed to lack fitness due to the extra energy demands imposed upon them by their introduced non-native genes. Thus, they may function effectively under optimized laboratory conditions but will fail once introduced into the harsh real-world environmental ecosystems they are intended to function within. The contained field release of strain HK44 was therefore established to answer two questions central to environmental bioreporter sensing, “Can a bioreporter such as HK44 serve as a continuous monitoring tool during a bioremediation process?” and “Can a genetically engineered strain like HK44 survive for extended periods in a natural ecosystem?” Strain HK44 was released into several large (4 m deep by 2.5 m diameter) contained lysimeter structures (Figure 6) that were packed with an artificially contaminated, aged soil comprised of a PAH mixture of naphthalene, anthracene, phenanthrene, and an Exxon Univolt 60 transformer oil. Bioluminescence from soil-borne HK44 populations was monitored over an approximate two year period using portable photomultiplier tubes inserted into the soil at various depths. Vapor phase naphthalene was monitored using biosensors consisting of alginate encapsulated HK44 cells interfaced with fiber optic cables. When challenged with naphthalene and minimal nutrient amendments, bioluminescence from HK44 populations could be detected directly within the soil matrix, and persisted for up to 30 day periods at intensities approaching maximums of approximately 5,000 photon counts s^−1^. The portable biosensors successfully profiled volatile naphthalene over short term periods, whereupon the alginate encapsulated cells would lose viability due to desiccation and would require replacement at weekly intervals. Encapsulation matrices that sustain long-term cell survival continue to be a challenge in biosensor applications, and there are clear needs for more advanced biomaterials capable of providing these requisite protective microenvironments. The survival of HK44 populations directly within the lysimeter soils, however, demonstrated surprising longevity. HK44 cells were quantified using viable plate counts on selective media and with a newly developed most-probable-number (MPN) method based on bioluminescence [63]. During the initial two year release, HK44 populations decreased from their initial inoculum of ∼1 × 10^6^ CFU·g^−1^ soil to a maintenance level of ∼1 × 10^3^ CFU·g^−1^ soil. Monitoring efforts continued four years post-release, and the HK44 microbes were shown to remain functionally active based on real-time *in situ* light production and recovery of *lux* mRNA from soil during metagenomic analysis of the extant microbial community, even though the selective pressure of the PAH contaminants of which it degrades was depleted at least two years prior. In preliminary sampling efforts now fourteen years post-release, HK44 cells no longer remained directly recoverable from the lysimeter soils, but the *luxA*, HK44-specific tetracycline resistance (*tetA*), and naphthalene dioxygenase (*nahA*) genes were identified by qPCR at low concentrations (∼100 to 2,000 copies·g^−1^ soil). Thus, it is apparent that genetically engineered microorganisms, despite a perceived lack of fitness, can survive for lengthy periods under real environmental pressures, and introduces new knowledge towards our understanding of risk assessment from a gene transfer perspective. Gene transfer refers to the exchange of genetic information among bacteria mediated by three classical methods; transformation, transduction, and conjugation. It represents one of the key driving forces to bacterial evolution and the rapid adaptation of bacterial species to environmental challenges. The introduction of recombinant genes into environmental ecosystems may contribute to gene transfer in unforeseen ways, and the lysimeter structures continue to serve as model ecosystems for studying these potential risks.

Strain HK44 also assisted in understanding plant-bacterial interactions during phytoremediation. Phytoremediation can be an effective method for cleaning up contaminated soils particularly if the contaminants are shallowly confined and available to plant roots. PAH contaminants, for example, show enhanced bioremediation in the presence of plant material likely due to microorganisms associated with the root rhizosphere. Bioluminescent induction from strain HK44 was used to investigate the microbial-plant interactions that potentially influence the expression of microbial genes responsible for PAH catabolism, under the hypothesis that the induction of the *nah* genes by root exudates correlated to increased biodegradation [43]. However, of 21 compounds tested, only three substrates released by plants induced bioluminescence in strain HK44; salicylate, acetylsalicylate, and methylsalicylate. None of the compounds associated with root extracts induced bioluminescence but rather inhibited bioluminescence at higher concentrations in the presence of naphthalene. However, HK44 populations associated with root extracts demonstrated more prolific growth and consequent increased levels of bioluminescence. Therefore, the initial hypothesis was rejected and the stimulation of microbial growth was concluded to be the main cause of enhanced biodegradation.

### 3.6. Monitoring of Immobilized Cell Viability and Physiology

The fusion of bioreporters with transducer elements to create biosensors requires that the integrated living cells be immobilized in microenvironments that promote long-term survival. However, the fundamental knowledge regarding immobilized cell physiology is somewhat lagging behind the applied research [38,84]. Bioluminescent microorganisms provide a simple method for monitoring the viability and physiological stressors that impact cells under immobilized states, with strain HK44 being applied in several such studies [52,57,70].

The viability and growth of strain HK44 immobilized into a silica sol-gel (tetramethoxysilane (TMOS) prepolymer) or composition silica sol-gel with alginate in various ratios was compared against similarly immobilized yeast (*Saccharomyces cerevisiae*) and plant (*Nicotiana tabacum*) cells. The HK44 cells were shown to be the most resistant to the adverse drying effects of the gels, and successfully emitted bioluminescence in silica-only encapsulants for up to one year. Performance in the silica/alginate mixtures was less optimal and prone to significant leakage of cells from the matrix. This leakage not only reduces sensitivity when applied as a biosensor transducer interface, but adds to the risk assessment potential of engineered bioreporter microorganisms that escape from their immobilized state into the environment. There is a clear need for improved and better characterized immobilization matrices before environmentally deployable biosensors can be readily adapted.

In another study, non-induced bioluminescence from strain HK44 was used as an indicator of stress imposed on the bacterial cells during encapsulation into silica sol-gel prepared from prepolymerized TMOS [57]. The release of methanol during this encapsulation process was hypothesized to act as a stressor affecting cell viability. Methanol was shown to affect non-induced bioluminescence as a function of concentration and time (increased bioluminescence in the presence of 1–7% methanol after 2–4 h, Figure 5). Immediately after mixing of the matrix with the cell suspension, bioluminescence was extinguished indicating significant encapsulation stress. Over the next 4 h, bioluminescence recovered (faster in thinner films) in accordance with decreasing concentrations of methanol in the film. Nevertheless, bioluminescence overestimated actual methanol concentration in the film. This observation led to the conclusion that methanol was the principal stress factor but likely not the only one.

### 3.7. Monitoring of Cell Concentration and Visualization of Transport Processes

Between 2001–2007, authors from Oregon State University and Pacific Northwest National Laboratory published a series of papers presenting HK44 as a useful tool for noninvasive quantitative measurement of bacterial growth in porous media. They used a CCD camera to follow the kinetics of induction of *lux* gene-dependent bioluminescence in HK44 in aqueous suspension and saturated or unsaturated translucent sand. Before O_2_ availability became a limiting factor, the rate of light emission (L) increased with the square of time (t) and linearly with increasing cell density (c). A nonlinear model that contains a constant B′ rate of increase in light emission, determined from the slope of a plot of $\sqrt{L/c}$ against t, predicted the behavior of *lux* induction in HK44 in sand. The kinetics of induction of light emission were similar regardless of the presence of glucose and the growth phase—stationary or log [67]. In a flat light transmission chamber filled with sand, temporal and spatial colonization was visualized by salicylate-inducible bioluminescence of HK44. Colonization expanded in all directions from the inoculation region, including upward migration against liquid flow. The portion of daily potential growth that remained within the chamber declined progressively, from 97% to 13%, between days 2 and 7. A limitation to using *lux*-based reporter systems for determination of bacterial growth in porous media is an underestimation of bacterial colonization in the interior of the colony due to oxygen limitation caused by rapid colony growth preventing *lux* gene induction. This development of “dark zones” might be employed in mapping of aerobic and anaerobic localities [77]. In cylindrical sand-filled columns, the bacterial-induced changes in the hydraulic properties were documented using non-induced HK44 [74]. Microbial colonization caused localized drying within the colonized zone, with decreases in saturation approaching 50% of antecedent values, and a 25% lowering of the capillary fringe height. Flow was retarded within the colonized zone and diverted around it concurrent with the expansion of the colonized zone between days 3 and 6 [75]. Similar studies of microbial transport in porous media were realized using the salicylate-induced bioluminescent bacterium *P. fluorescens* 5RL. In contrast to previously shown studies with HK44, induced 5RL cells were in a grain filled chamber with non-growth promoting media with salicylate. Nevertheless, the results confirmed that with adequate amounts of bacteria and salicylate, the limiting factor of light emission is oxygen bioavailability [85].

## 4. Strains Related to *P. fluorescens* HK44

This review would not be complete without a brief overview of related bacterial strains that contributed to our understanding of bioreporters in general as well as strain HK44 in particular. Currently, a large number of bioluminescent bioreporters are available enabling bioluminescent monitoring of various metabolic pathways, stressors and physiological changes, and external factors including pollutants and other chemical mediators. Despite the majority of bioreporters being constructed within engineered strains of *Escherichia coli*, a significant number are based upon bacteria isolated from soil and water, which enables more relevant environmental application [16]. Among others, a series of engineered Pseudomonads including strains of *P. fluorescens* are available that express *luxCDABE* constitutively [65] as well as upon induction by priority pollutants such as aromatic and aliphatic hydrocarbons, chlorinated ethenes, and heavy metals. An excellent recent review is available listing these strains and summarizing their applications in the detection of water contaminants [16].

The strain most related to HK44 is the engineered bioluminescent strain *P. fluorescens* 5RL, which served as the original parent for plasmid pUTK21. Strain 5RL lacks the ability to degrade salicylate because its sole copy of the salicylate operon is inactivated by insertion of the *luxCDABE* gene cassette within *nahG*. While such inactivation was disadvantageous for biodegradation proposes (later overcome by construction of strain HK44), it was beneficial for other applications. For example, strain 5RL (plus several other naphthalene degrading strains including the antecedent strain 5R) was used to study the transformation of substituted naphthalenes and other PAHs by the upper naphthalene pathway [82,86,87]. Salicylate serves as an inducer of bioluminescence in strain 5RL and, since it is not degraded, bioluminescence output can be sustained at fairly high levels. This capability was used in several visualization and colonization studies, including the development of bioluminescent biosensors [88–92], ecological studies of microbial communities and rhizosphere interactions [93–96], monitoring of bacterial colonization of dental root-canals and improvement of their mechanical removal [97–100], and visualization of bacterial transport in porous media [85].

## 5. Conclusions and Future Perspectives

This review summarizes the broad range of studies carried out with the bioluminescent bioreporter *P. fluorescens* HK44 since its introduction in 1990. The indigenous nature of strain HK44 in combination with target inducible bioluminescence has enabled its service in a series of bio-analytical applications for the detection of PAH and other chemical targets in soil, water, and air. However, the functionality of bioreporters in terms of detection limits, broad group selectivity, and potential interferences demonstrates that bioreporter assays are not replacements for standard chemical analytical methods, but rather complement them through speed of response, economical cost, and an ability to survey for bioavailability. Regrettably, relatively few applications using HK44 and other bioreporters are being implemented within authentic ecosystems, primarily due to the legislative restrictions encompassing the use of genetically engineered microorganisms and their environmental release. Also, past work with bioreporters typically focused on bioluminescent responses to single chemicals rather than more true-to-life complex admixtures, although this is changing as scientists begin to incorporate pattern learning algorithms and decision tree models to identify multiple chemicals via unique “fingerprint” light emission profiles [101,102]. Nevertheless, the studies that have been performed have accumulated invaluable data concerning the competence of bioreporters in targeted biosensing, their fitness under environmental pressures, and the risks they potentially incur upon environmental release. With the recent sequencing of strain HK44, even more robust characterizations can now be performed to better elucidate and understand the fate, activities, and interactions of this strain under real world scenarios to stimulate and enable a broader scope of future full-scale field applications or, perhaps even more interestingly, to retroactively re-examine and model previous field and microcosm trials to more fully comprehend the true fitness and fate of recombinant microorganisms.

## Figures and Tables

**Table 1.** Recommended reviews and book chapters closely related to this review.

**Topic**	**Reference**
**Bioreporters in general**	
Design and applications of reporter bacteria	[2,6]
Reporter proteins in whole-cell optical bioreporter detection systems	[3,7]

**Environmental applications of bioreporters**	
Application of microbial bioreporters in environmental microbiology and bioremediation	[8]
Gene expression monitoring in soils by mRNA analysis and gene *lux* fusions	[9]
Environmental monitoring using whole-cell bioreporters	[1,10–13]
Field applications of genetically engineered microorganisms for bioremediation processes	[14]
Reporter gene bioassays	[15]
Practical considerations of environmental applications of bioreporters	[16,17]

**Whole-cell biosensors**	
Microbial bioreporter sensing technologies for chemical and biological detection	[18]
Whole-cell aquatic biosensors	[19]
Microbial biosensors	[20–22]
Bioluminescent-bioreporter circuits	[23]

**Bioluminescence**	
Bacterial bioluminescence, genetics, biochemistry, control	[24–28]

**PAH pollution and metabolism**	
PAH biodegradation and bioremediation	[29,30]
Metabolism of naphthalene in bacteria	[31,32]
Promoters of PAH degradation pathways	[33]

**Biodegradation/bioremediation**	
Bioremediation technologies	[34–37]

**Table 2.** Bacterial strains and plasmids used to construct *P. fluorescens* HK44.

**Bacterial strain**	**Strain derivation**	**Plasmid**	**Genotype**
*P. fluorescens* 5R	Wild-type	pKA1	*nah^+^sal^+^lux*^−^
*P. fluorescens* 5RL	Tn*4331*	pUTK21	*nah^+^sal*^−^*lux^+^*
*P. fluorescens* 18H	Wild-type	Cryptic	*nah*^−^*sal^+^lux*^−^
*P. fluorescens* HK44	18H × 5RL	pUTK21	*nah^+^sal^+^lux^+^*

**Table 3.** Biosensing applications and related studies that have utilized *P. fluorescens* HK44 as a bioreporter.

**Purpose**	**Experimental**	**Results and conclusions**	**Ref. Year**
Construction of *nah-lux* gene fusion plasmid pUTK21 bioreporters *P. fluorescens* HK44 and 5RL	Chemostat (MM with succinate, tetracycline and yeast extract), 2.5 h retention time, addition of NAP to influent Batch test with slurries: 1:1 HK44 suspension (10^9^ cells·g^−1^ in phosphate buffer) and soil	Construction of pUTK21 plasmid and HK44 strain. Repetitive exposure to NAP induced repeatable BL responses. BL response in slurries lower due to light quenching.	[4] 1990
Biosensor for naphthalene and salicylate	Cells grown in batch cultures and exposed to MM plus SAL and NAP, or spiked soil slurries.	Demonstrated linear responses between NAP (up to ∼3.25 mg·L^−1^) or SAL (up to ∼20 mg·L^−1^) concentrations and BL. LOD (NAP) 45 μg·L^−1^. Higher specific BL responses were achieved in exponential cultures than in resting cultures.	[48] 1992
Detection of hydrocarbons in soil samples	Three bioreporters including HK44. Cell suspension in MM mixed with soil (1:1) and BL measured.	BL discrimination of contaminated and uncontaminated samples.	[60] 1994
Biosensor for continuous monitoring of NAP and SAL bioavailability	Cells immobilized in Sr-alginate on an optical light guide. BL measured continuously using a sample stream consisting of a maintenance media with oxygen, nutrients and carbon substrates.	NAP and SAL in sample stream resulted in fast (2 min) repeatable increase of BL. Low to no BL responses detected to non-target substrates (glucose, toluene). Positive BL responses to environmental samples (leachates of manufactured gas plant polluted soil or jet fuel).	[51] 1994
Kinetics of BL response of encapsulated HK44	Used a packed-bed reactor containing HK44 cells encapsulated in Ca-alginate and a photodiode. Cells were exposed to media with NAP, SAL and glucose.	SAL had low sorption to alginate but NAP had significant sorption onto alginate. First order biodegradation observed for both SAL and NAP with a higher magnitude response for NAP than SAL. A mathematical model was developed for BL responses and biodegradation.	[39] 1997
Repetitive bioluminescent response and survival of alginate-encapsulated HK44 under various pH and nutrient availability	Ca-alginate encapsulated cells exposed to 100 mg·L^−1^ SAL under various pH (3–7) and nutrient availability (simple solution—SAL in water, complex solution—SAL in YEP) and simulated groundwater	Most stable BL responses at pH 6, no response at pH < 6. CFU declined at pH < 6. ∼50% of added SAL was degraded within 5 h.	[53] 1997
Response of HK44 to fuel extracts and evaluation of interactions of toxicants	Exponential culture cultivated in YEPG. Resting culture in MM. Sample: bacteria 1:1 Water extracts from contaminated soil Aqueous solutions of JP-4 jet fuel, solvents, cyanides, and heavy metals.	BL assay coincided with but overestimated NAP concentration in soil. Solvents increased bioluminescence without induction of *nah-lux* genes but only in growing cultures. A synergistic effect occurred between NAP and solvents on BL. BL decreased upon exposure to toxic compounds (heavy metals, cyanides).	[54] 1998
Monitoring of HK44 escape during loading of long-term lysimeter soil experiment	Anderson air sampler, gravity sampling from air. Enumeration of HK44 using selective plating, later induced by naphthalene and counted in the dark	Rare escape of HK44 from lysimeter, higher numbers at lower humidity and lower wind.	[61] 1999
Controlled field release of strain HK44 for bioremediation process monitoring and control	Cells added to soils contained in 6 lysimeters and monitored by CFU on YEPSS. Photon counting used to monitor BL directly in the lysimeter using a fiber optic cable and in soil samples using a photomultiplier module.	HK44 cells survived for over 660 days in both uncontaminated and hydrocarbon-contaminated soil. BL also detected in the presence of soil hydrocarbons over the 2-year period.	[62] 2000
Development of MPN method for quantification of bioluminescent bioreporters in soil	1:2 dilutions of soil in saline made in microtiter plates. A 6 mg·L^−1^ SAL solution added to induce BL. Plates incubated for 16 h and BL measured.	*lux*-based MPN method more accurate than selective plating on YEPSS and provided a good ecological assessment of HK44 population dynamics over 474 days.	[63] 2000
A model to quantify cell density in translucent porous media	Cells grown to stationary phase in a MM containing glucose + TC. Cell suspensions 10^5^–10^8^ cells·mL^−1^ in aqueous solution or sand. BL induced by addition of SAL.	A nonlinear model developed containing a rate of light emission constant. The model was used to predict light induction under variety of conditions.	[64] 2001
Comparison of responses of 6 bioluminescent bioreporters to bioremediation of five contrasting oils	Tested five petroleum oils (light-heavy) containing predominantly aliphatic hydrocarbons. Tested three metabolic (constitutive) bioreporters and three catabolic (inducible) bioreporters including HK44.	BL response of HK44 negligible in comparison to aliphatic hydrocarbon-specific bioreporters. Organic solvents increased bioluminescence of HK44.	[58] 2001
Development of quantitative structure activity relationship (QSAR) model for biotransformation and toxicity	HK44 used to monitor biotransformation and a constitutive *P. fluorescens* bioreporter to monitor toxicity of one ring (six compounds), two ring (seven compounds), three ring (five compounds) and four ring (two compounds) PAHs.	QSAR model based on BL response of HK44 and toxicity based on BL in *P. fluorescens*. Toxicity was predictable by log P_kow_; biotransformation was predictable by lowest unoccupied molecular orbital (LUMO) energies.	[65] 2001
Monitoring of vertical transport of a field-released HK44 through soil	Water table manipulated in two lysimeters. One lysimeter sprayed with a 1.5 × 10^7^ cells·mL^−1^. HK44 transport in groundwater was quantified by CFU on YEPSS + TC and colony hybridization.	Significant transport of HK44 60 cm below inoculation area in response to groundwater fluctuation.	[66] 2001
Noninvasive quantitative measurement of bacterial growth in porous media under unsaturated-flow conditions to monitor hydrology-microbiology interactions	HK44 grown in nitrate-free MM with glucose. In chamber experiments containing quartz sand and the glucose concentration was reduced and salicylate was periodically added as an inducer of BL.	HK44 growth predicted over 4 orders of magnitude using a nonlinear model based on salicylate induced bioluminescence. HK44 readily colonized the sand and expanded in all directions even against the liquid flow.	[67] 2002
Considerations for modeling bacterial-induced changes in hydraulic properties of variably saturated porous media	Packed sand columns with steady unsaturated flow conditions. Monitored HK44 growth over 1 week with a steady flux of MM media with glucose.	Reviews liquid-saturated porous media system models and discusses characteristics important for modeling unsaturated porous media systems.	[68] 2002
Determination of the kinetic parameters of bacterial luminescence	Batch, turbidostat, and chemostat cultivation of HK44 in MM + SAL.	Determination of the kinetic constants of bioluminescence. Increased loss of *lux* phenotype during long-term cultivation.	[50] 2003
Detection of PAHs in marine sediments	HK44 in YEPG (OD_600_ = 1) mixed 1:1 with MM extract from sediment sample. BL measured after 9 h.	Sigmoidal NAP calibration curve. BL induced by uncontaminated sediment comparable to negative control. Significant overestimation of PAH concentration in contaminated sediment compared to gas chromatography.	[69] 2003
Revealing increased PAH biodegradation in rhizosphere	HK44 BL response to compounds (50 mg·L^−1^) present in root exudates and complete exudates compared to BL of HK44 grown on YEPSS.	BL and NAH degradation increased in the presence of root extracts. BL response induced by exudates was predominantly lower than for SAL suggesting that increased BL in the presence of root extracts was due to increased bacterial growth rather than specific induction of the naphthalene degradative pathway.	[43] 2004
Monitoring of viability of encapsulated cells (yeast, bacteria, and plant cells)	HK44 cultivation in LB, encapsulated into silica and silica-alginate matrix (sol-gel)	Lower viability in denser films, alginate increases viability but causes cell leakage. Bacteria (HK44) most resilient to encapsulation.	[70] 2004
Flow-through biosensor for detection of low concentrations of NAP	Cells cultivated in YEPG + TC. Samples containing buffer, NAP and cells injected into the flow cell.	Optimal cell concentration 0.2 g·L^−1^. Response time 19 min, maximum BL after 35 to 40 min. A linear response detected up to 0.4 mg·L^−1^ NAP with LOD of 0.02 mg·L^−1^.	[71] 2004
Application of flow-through sensor for detection of low NAP concentrations in wastewater samples	Stationary-phase cells. Real waters (influent and effluent into biological water treatment) mixed with HC at 2.4 mg·L^−1^ and 1.1 mg·L^−1^	Decrease of specific BL at higher HC concentrations in real waters due to toxicity. 15 mg·L^−1^ nitrates doubled specific BL, ≥30 mg·L^−1^ nitrates caused BL decrease.	[72] 2004
Coupling of non-exhaustive extraction technique (NEET) with NAP mineralization	Hydroxypropyl-β-cyclodextrin (HPCD) and amberlite XAD-4 tested for the ability to extract soil-bound NAP. HK44 grown in YEPSS and used as biosensor. ^14^C- NAP used to evaluate mineralization.	NAP mineralization highly correlated with extractable fraction of HPCD but not XAD-4. HK44 BL correlated with NAP concentrations in the NEET extracts.	[73] 2004
Experimental observations and numerical modeling of coupled microbial and transport processes in variably saturated sand	Sand columns inoculated with HK44. Liquid provided as MM with glucose. BL induced with MM + SAL	Determined attachment and detachment rate coefficients for use in fully coupled multi-fluid flow equations.	[74] 2005
SAL biosensor	Cells grown in batch in LB + TC. Sol-gel encapsulation (10^7^ cells·g^−1^). Induction in YEPS.	BL repeatable ±20%, saturation-type CR, LDC_nap_ 1.2 mg·L^−1^, LDC_sal_ 0.05 mg·L^−1^. Maximum BL after ∼4.5 h, lag 50 min, films with ≤10^7^ cells·g^−1^ stable for >8 months and 50 induction cycles. Response to 4 SAL analogs, however with higher LDC.	[52] 2005
Impact of microbial growth on water flow and solute transport in unsaturated porous media	Silica sand columns inoculated with HK44 in MM + glucose. BL induced with SAL in MM. Bromophenol blue dye solution used to measure flow paths.	Real-time noninvasive measurements in porous media using SAL induced BL. Followed growth and transport of HK44. Significant impact of bacterial growth on water retention.	[75] 2006
Selectivity of HK44 BL response	HK44 cultivated in LB + TC, induction in YEPS, batch, stationary culture, 95 tested compounds	BL response to 45 analogs of NAP, SAL, and PAHs. Response to analogs generally lower.	[55] 2007
Salicylic acid degradation from aqueous solutions—basic data for construction of SAL biosensor	Free stationary culture, 0.1–1.0 g·L^−1^·cell concentration. Water solutions (25–200 mg·L^−1^ SAL) with/without yeast-extract (0.01%)	Maximum degradation rate after 30 min. Yeast extract improved biodegradation but prolonged lag. Higher degradation rate at lower SAL concentrations. Higher specific degradation rate at lower cell densities (3 hypotheses).	[76] 2007
Model of the colonization dynamics in variably saturated, translucent quartz sand	Silica sand columns inoculated with HK44 in MM + glucose. BL induced with SAL. Bromophenol blue dye solution used to measure flow.	In 6 days the colonized region expanded 15 cm laterally and 7–8 cm upward against the flow. Apparent water saturation and capillary fringe also decreased. Numerical model developed to account for coupled flow, reactive transport and biological processes.	[77] 2007
Biosensor of NAP in air	Air with flow-through. HK44 encapsulated in fresh 2% agar + 0.01% yeast extract, 10^7^ cells·g^−1^ late exponential phase	LOD 20 nmol·L^−1^ (below threshold for air), LR 50–260 nmol·L^−1^ Maximum BL after ∼80 min, 1ag 30 min. Response dependent on NAP concentration rather than flow-rate.	[78] 2008
Correlation of NAP biodegradation in historically contaminated soil with non-exhaustive extraction techniques	BL responses to non-exhaustive solvent extraction (water, cyclodextrin, methanol) of spiked soil and contaminated soil. Comparison to biodegradation by *P. fluorescens* TTC1 (1 month).	Linear response to NAP in extracts up to ∼800 mg·g^−1^ soil, slope dependent on the extraction technique. Cyclodextrin extraction predicted biodegradation most accurately.	[79] 2009
Predicting bioremediation and bioavailability of HC in soil for field studies	5 bioassays with BL bioreporters including HK44. HK44 in saline mixed with methanol extracts of soil.	BL assays enabled good prediction of bioremediation. Field bioremediation ∼3 times slower.	[80] 2009
Automatic formation of structure-induction/toxicity hypotheses of NAP analogs	HK44 grown in LB + TC, stationary culture. Induction in YEPS, batch	7 SAR hypotheses automatically generated. Out of 12 NAP analogs 10 induced BL in HK44 supporting 3 hypotheses and rejecting one.	[56] 2010
Monitoring of encapsulation stress	HK44 cultivated in LB + TC, stationary culture. Encapsulated into silica matrix (sol-gel)	Evolving methanol is the principal stress factor, however not sole factor. Stress proportional to film width.	[57] 2010

Abbr.: BL—Bioluminescence, CR—Concentration-response, CFU—colony forming units, HC—hydrocarbons, LB—Luria broth, LDC—Lowest detectable concentration, LOD—Limit of detection, LR—linear response, MM—mineral medium [48], MPN—most probable number, NAP—naphthalene, OD—optical density, SAL—salicylate, SAR—structure-activity relationship, TC—tetracycline, YEP—Yeast extract-polypeptone medium [53], YEPS—Yeast extract-Peptone-Succinate medium [52,55,56,70], YEPG—Yeast extract-peptone-glucose medium [71].
